# Supplementary figures and images for: RetiGene, a comprehensive gene atlas for inherited retinal diseases (IRDs)
Source: bioRxiv. 2025 Jun 8:2025.06.08.653722. Preprint. [Version 1] doi: 10.1101/2025.06.08.653722 (PMC12259000; doi:10.1101/2025.06.08.653722)

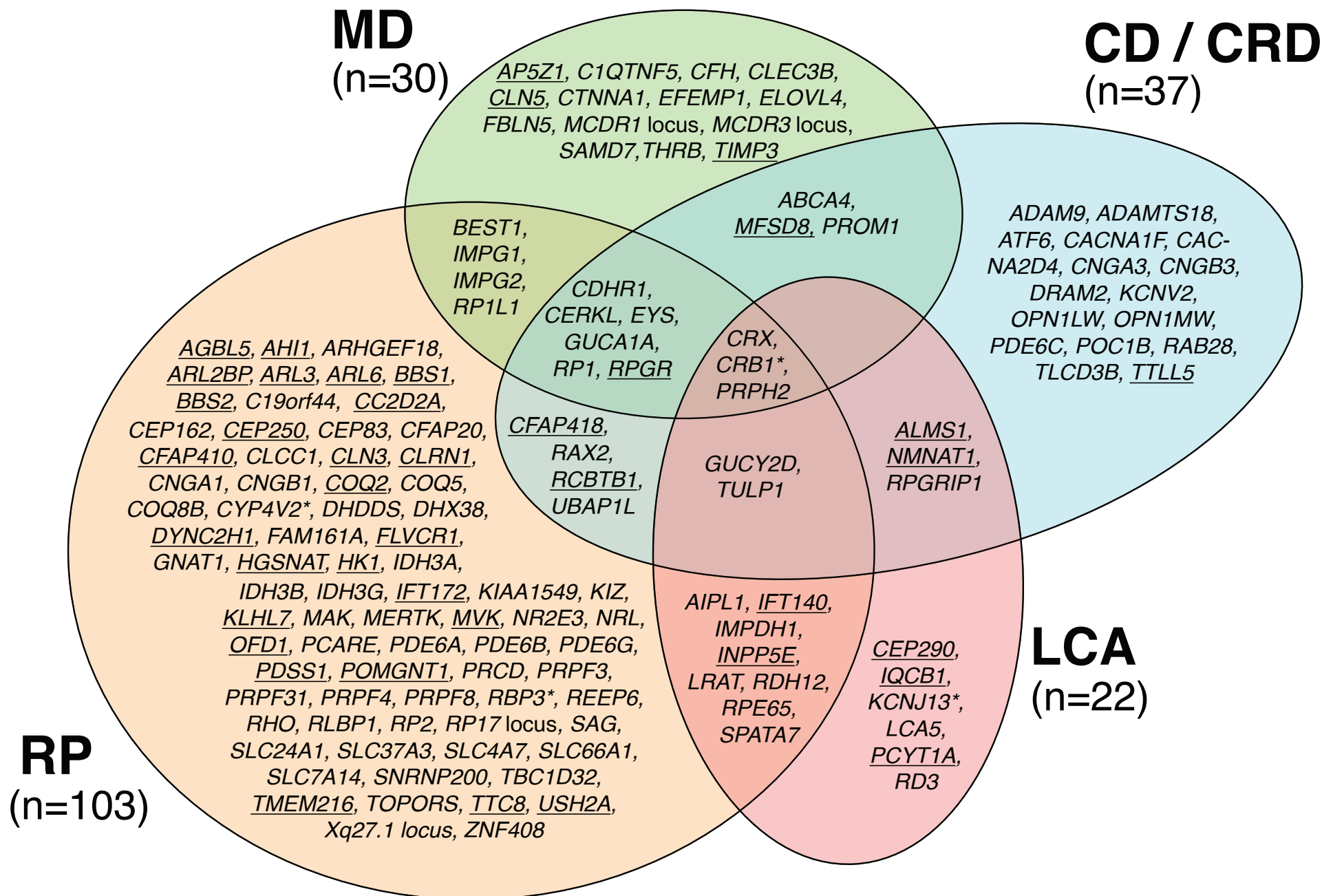

Supplement: Supplement 1 — Figure S1: Venn diagram of genes and loci associated with the most common non-syndromic IRDs. Underlined genes are linked to both non-syndromic and syndromic phenotypes. Asterisks point to genes that can also be involved in non-retinal ocular diseases. [file media-1.pdf]

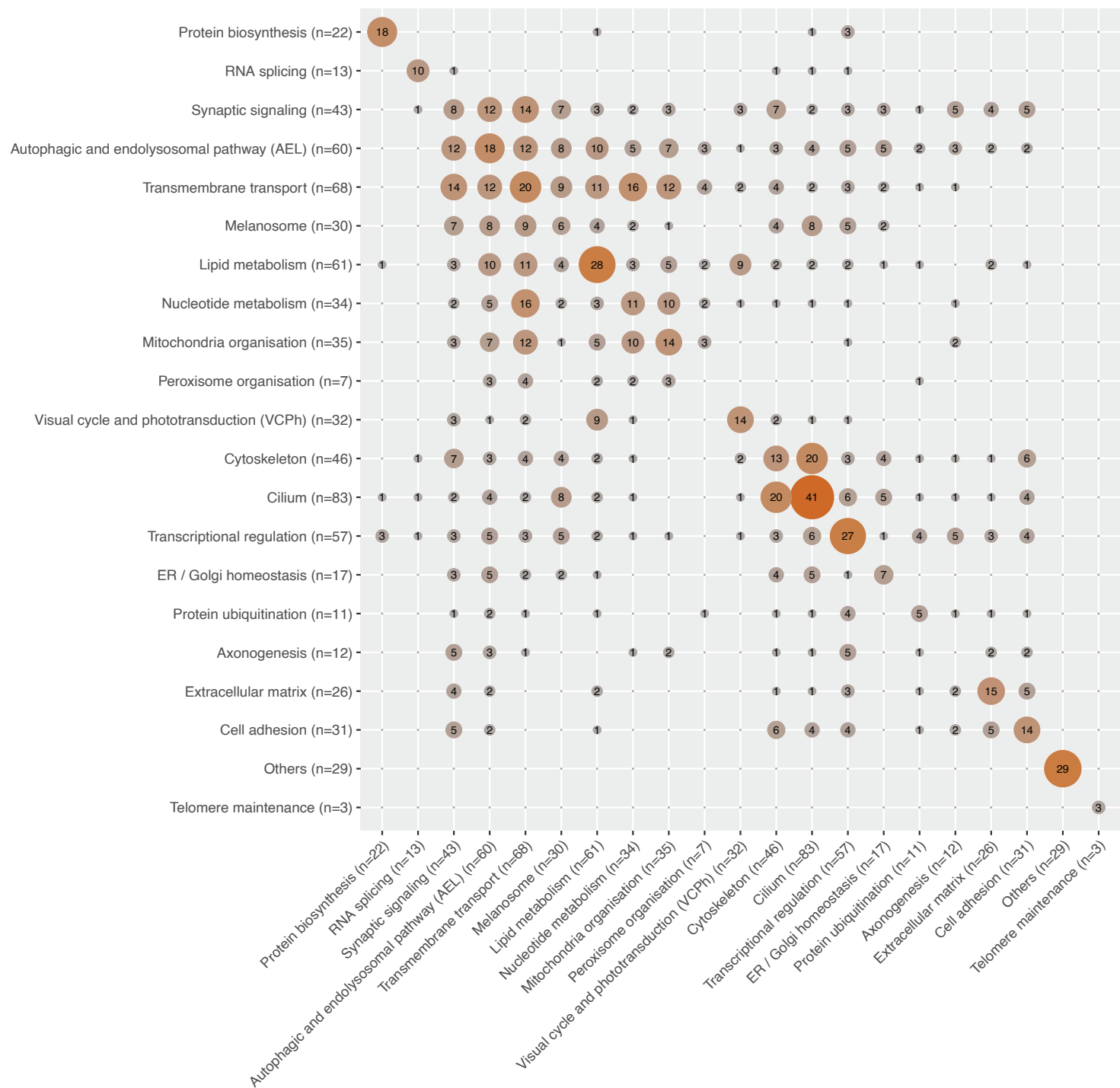

Supplementary Figure 2

Supplement: Supplement 2 — Figure S2. Overlap among functional categories associated with IRD genes. Functional categories are listed along both axes. n (or plain numbers), number of genes. [file media-2.pdf]

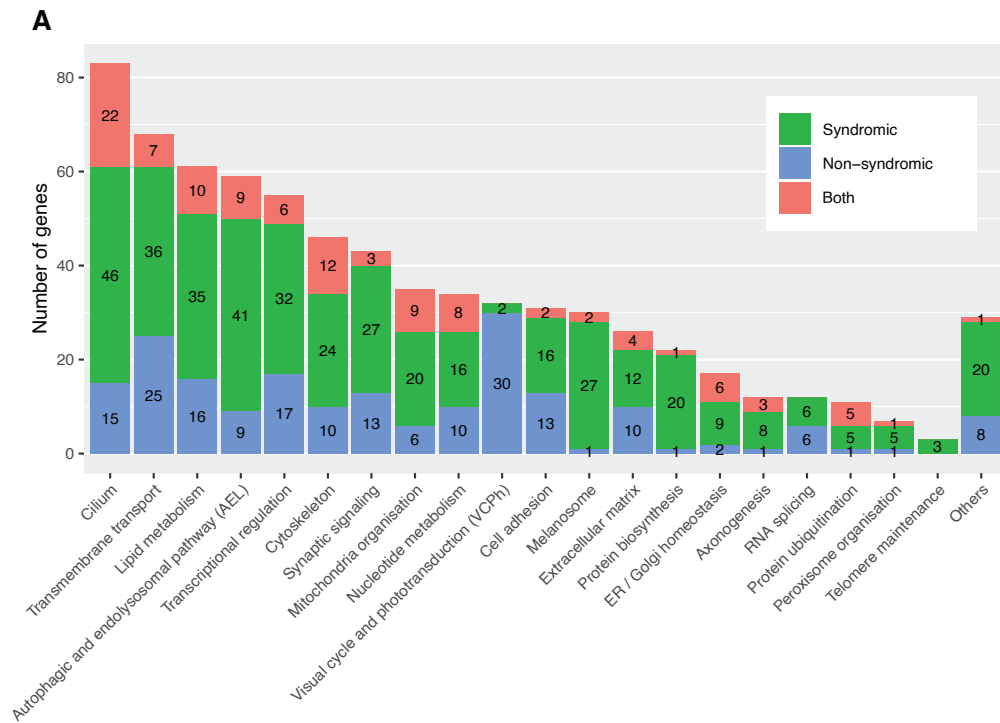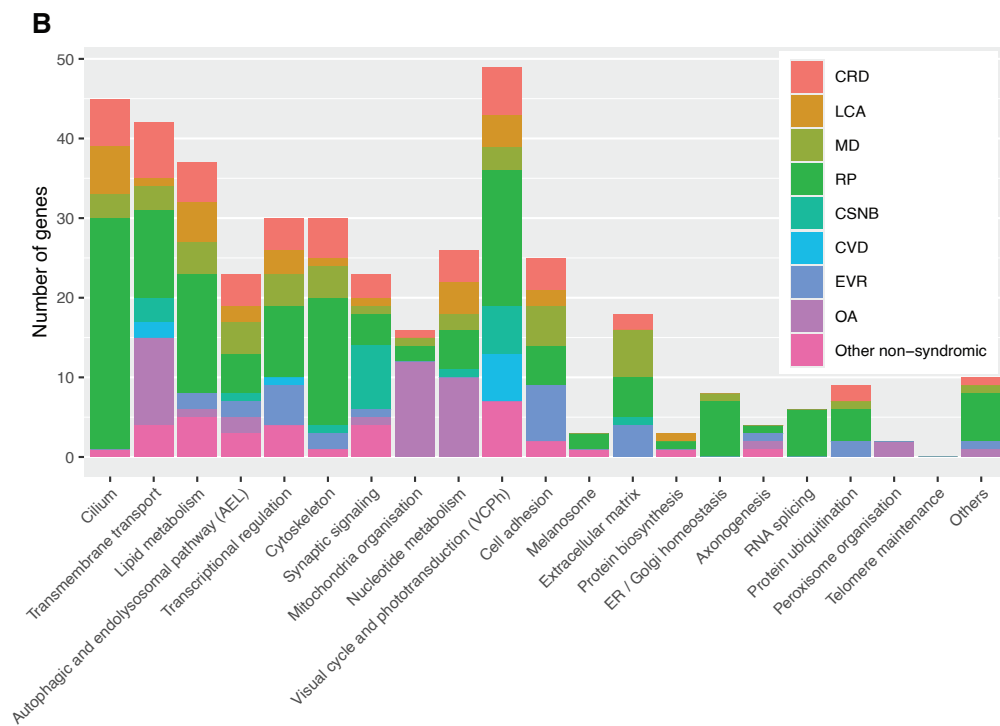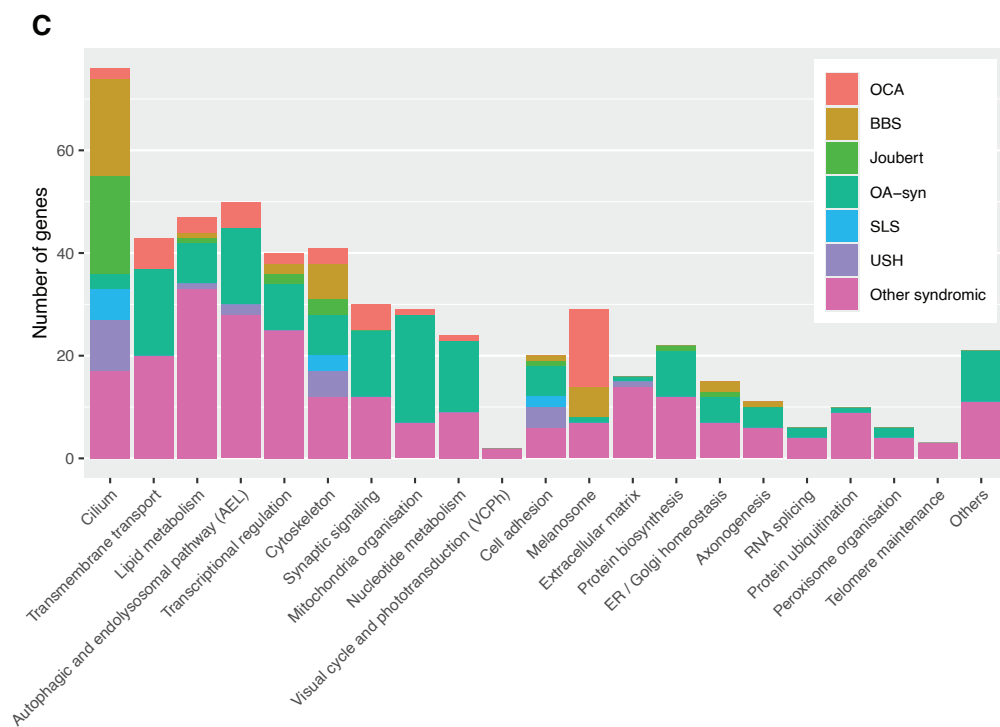

Supplement: Supplement 3 — Figure S3: Functional classification of genes, stratified by phenotypes. (A) Broad phenotypic categories. (B) Non-syndromic phenotypes. (C) Syndromic phenotypes. [file media-3.pdf]

**A**

Phenotype category

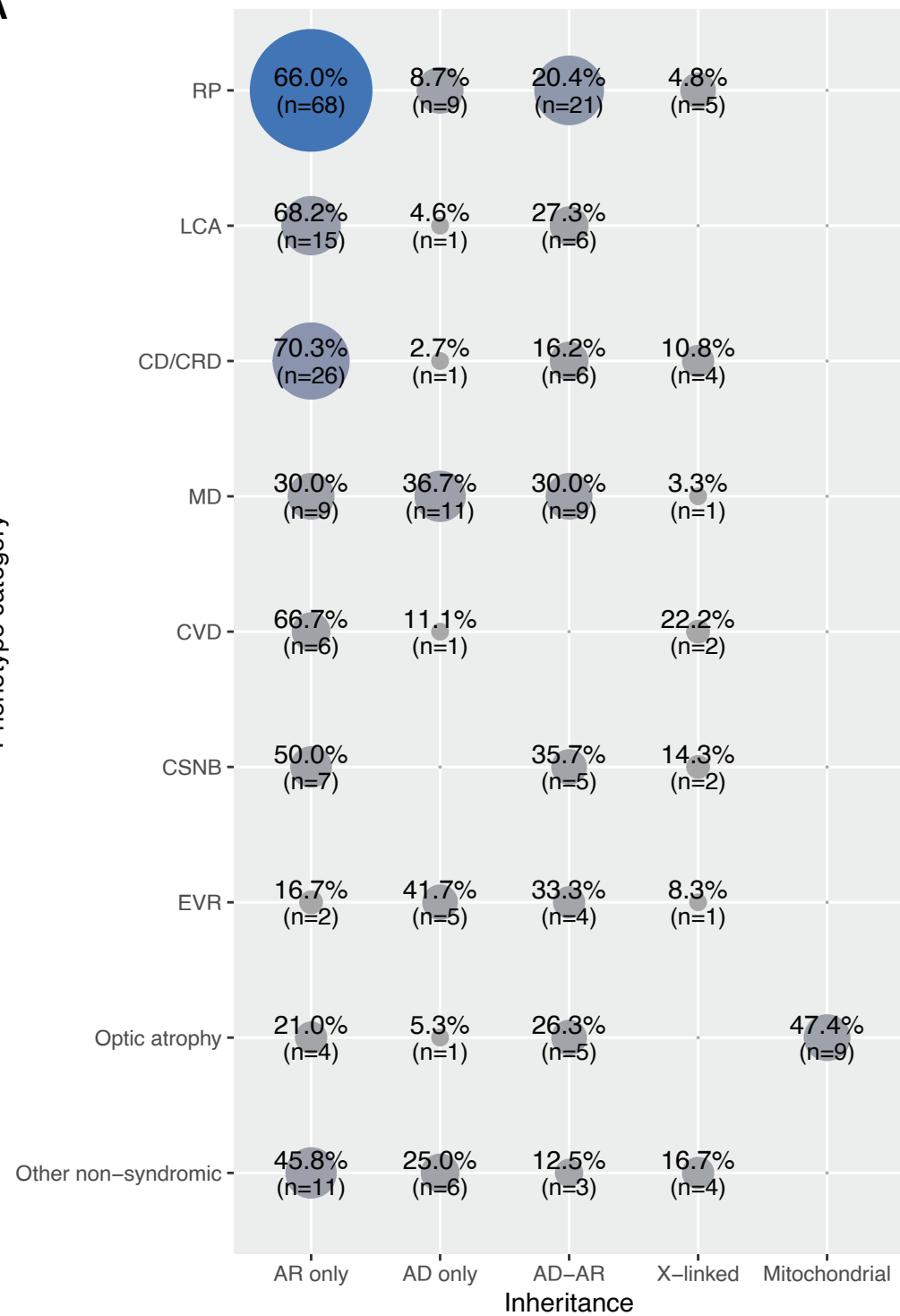**B**

Phenotype category

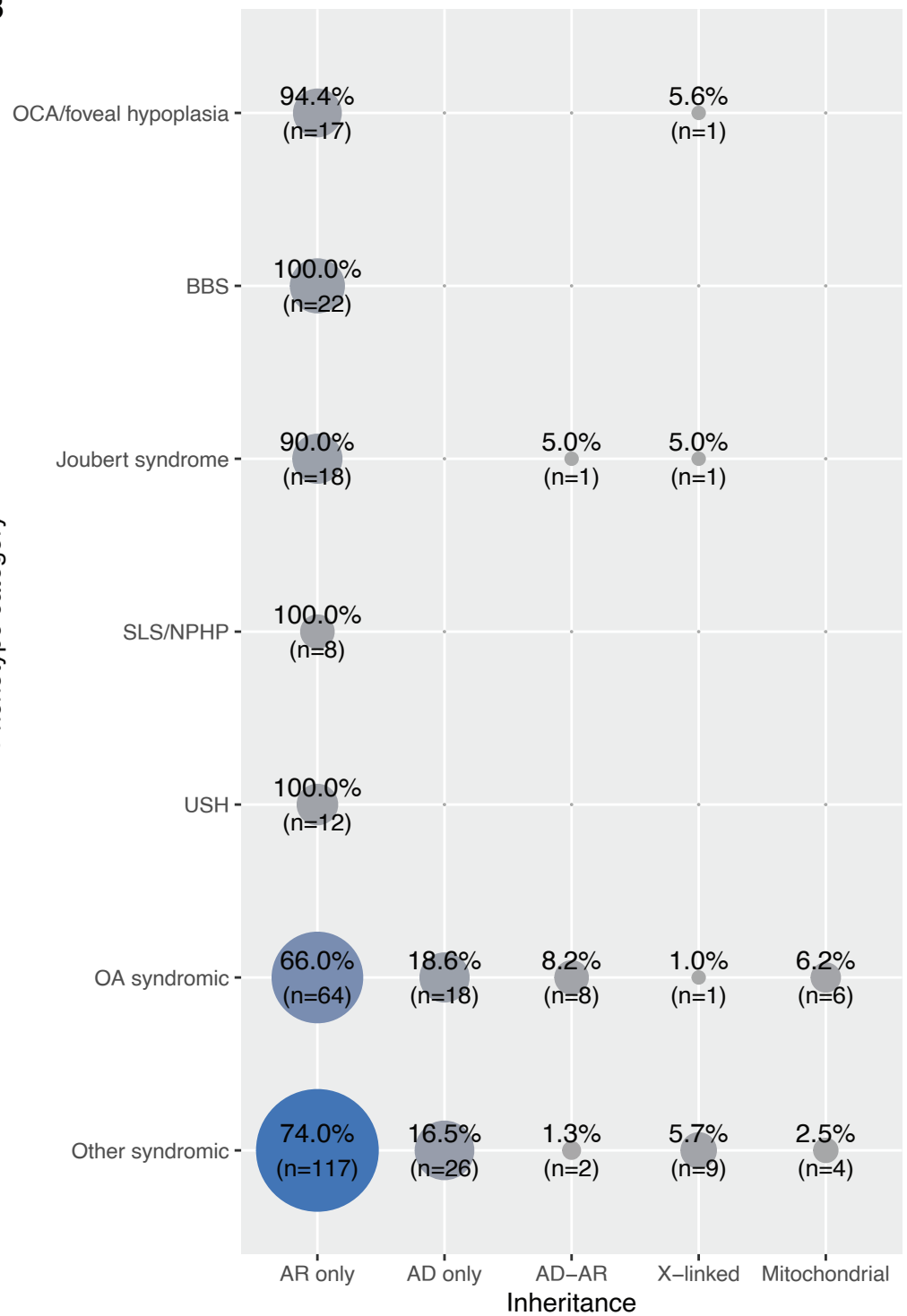

Supplement: Supplement 4 — Figure S4: Co-occurrence matrix between phenotypes and their inheritance. (A) Non-syndromic phenotypes. (B) Syndromic phenotypes. n, number of genes. [file media-4.pdf]

**A**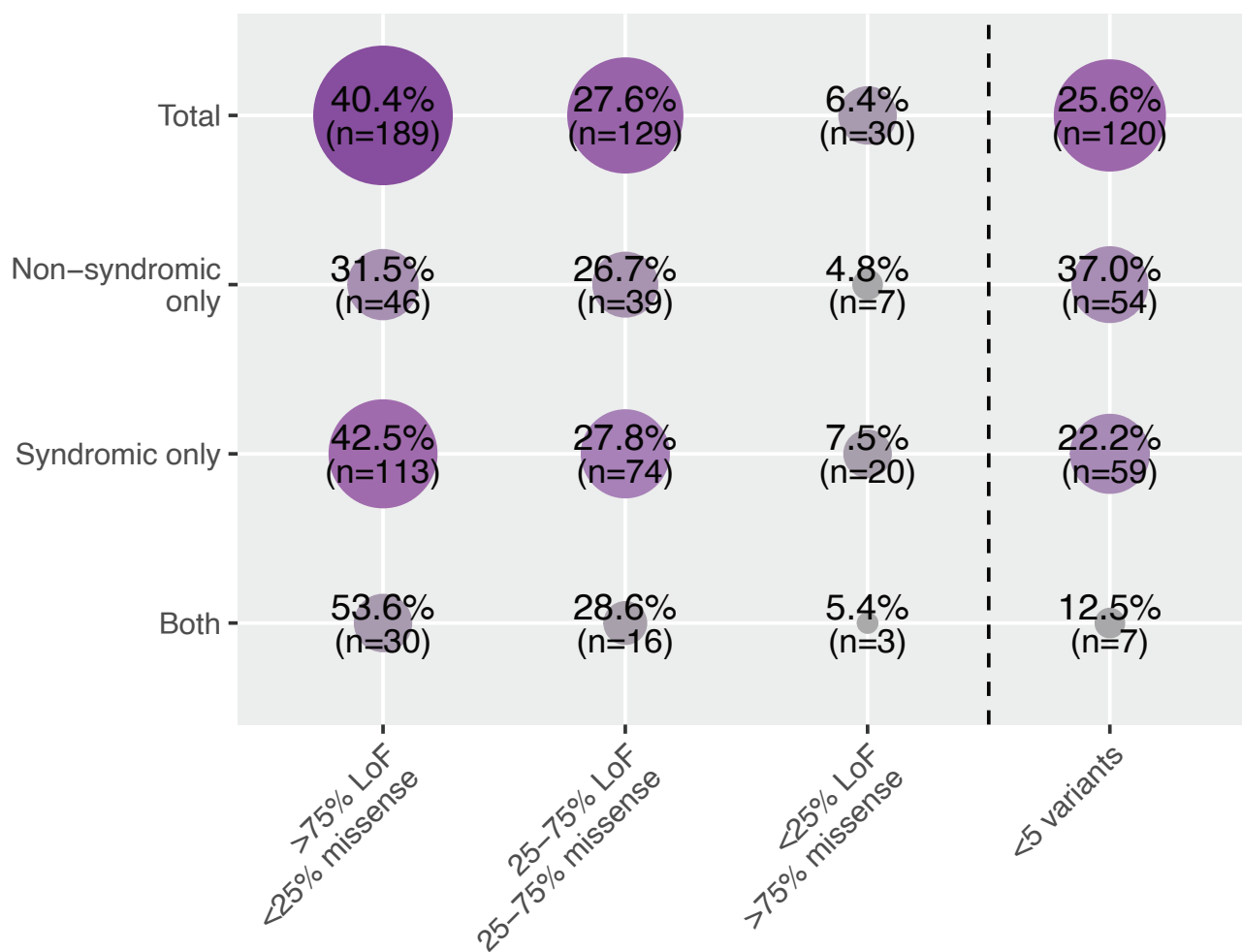**B**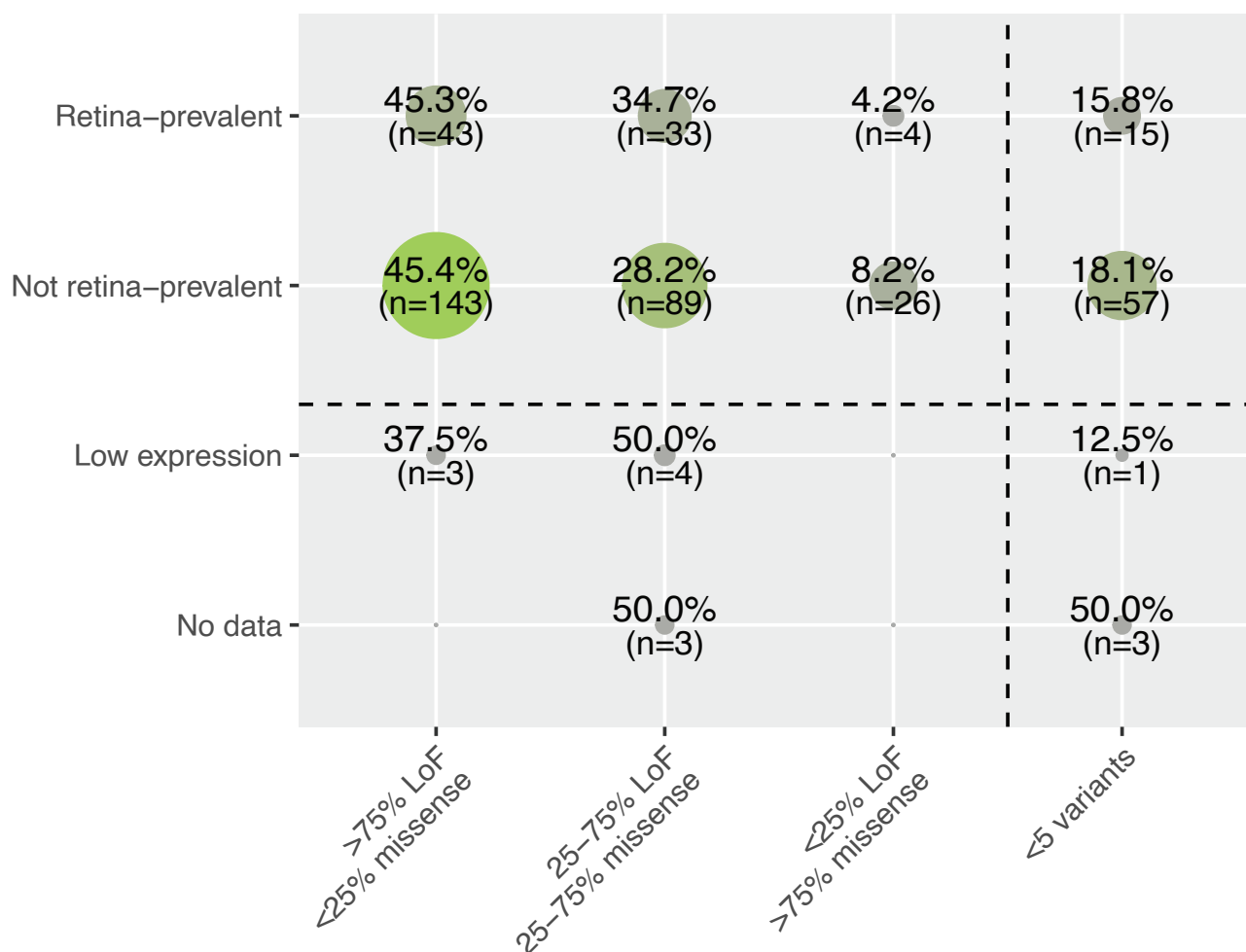

Supplement: Supplement 5 — Figure S5: Co-occurrence matrices between types of pathogenic variants and (A) broad phenotypic categories or (B) specific tissue expression from bulk RNA-Seq. n, number of genes. [file media-5.pdf]

A

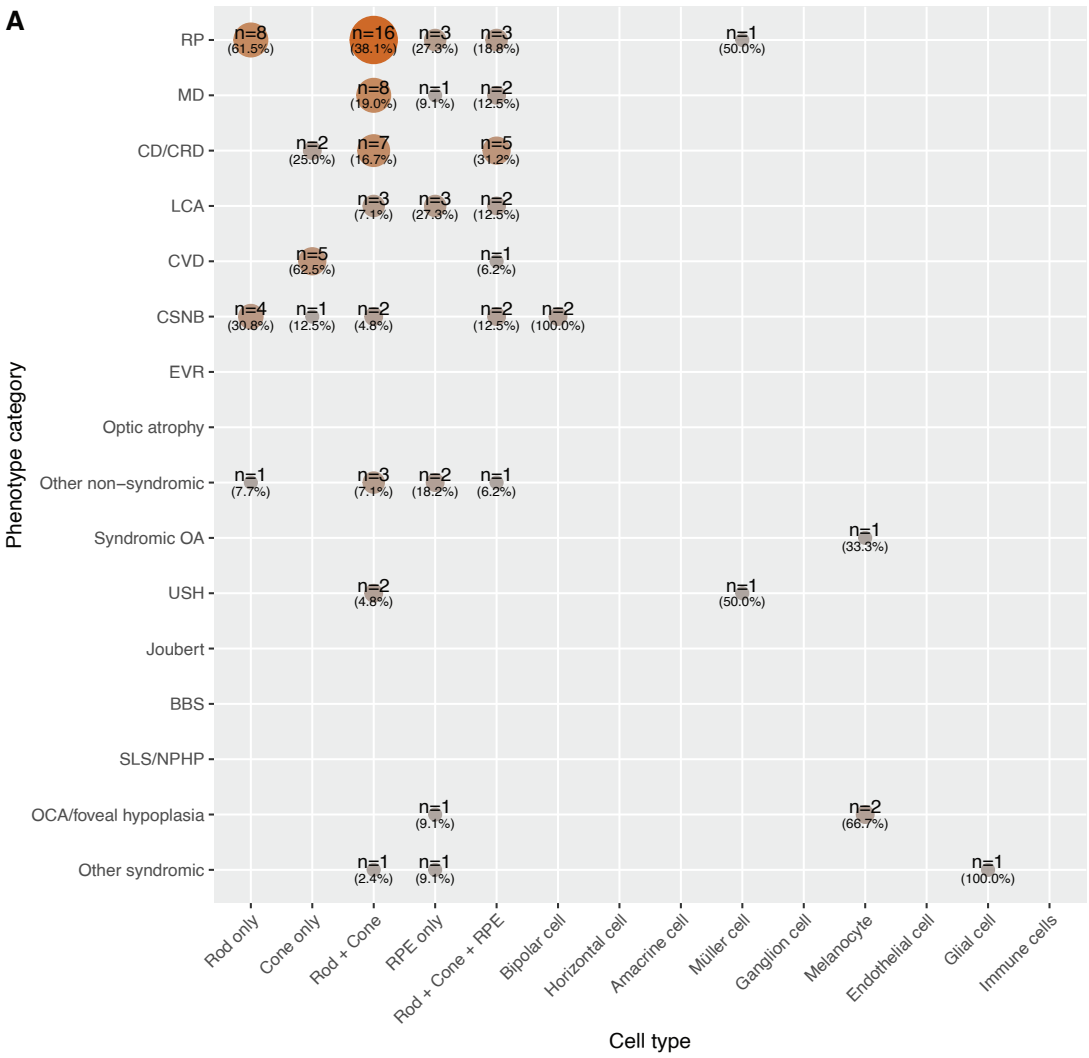

B

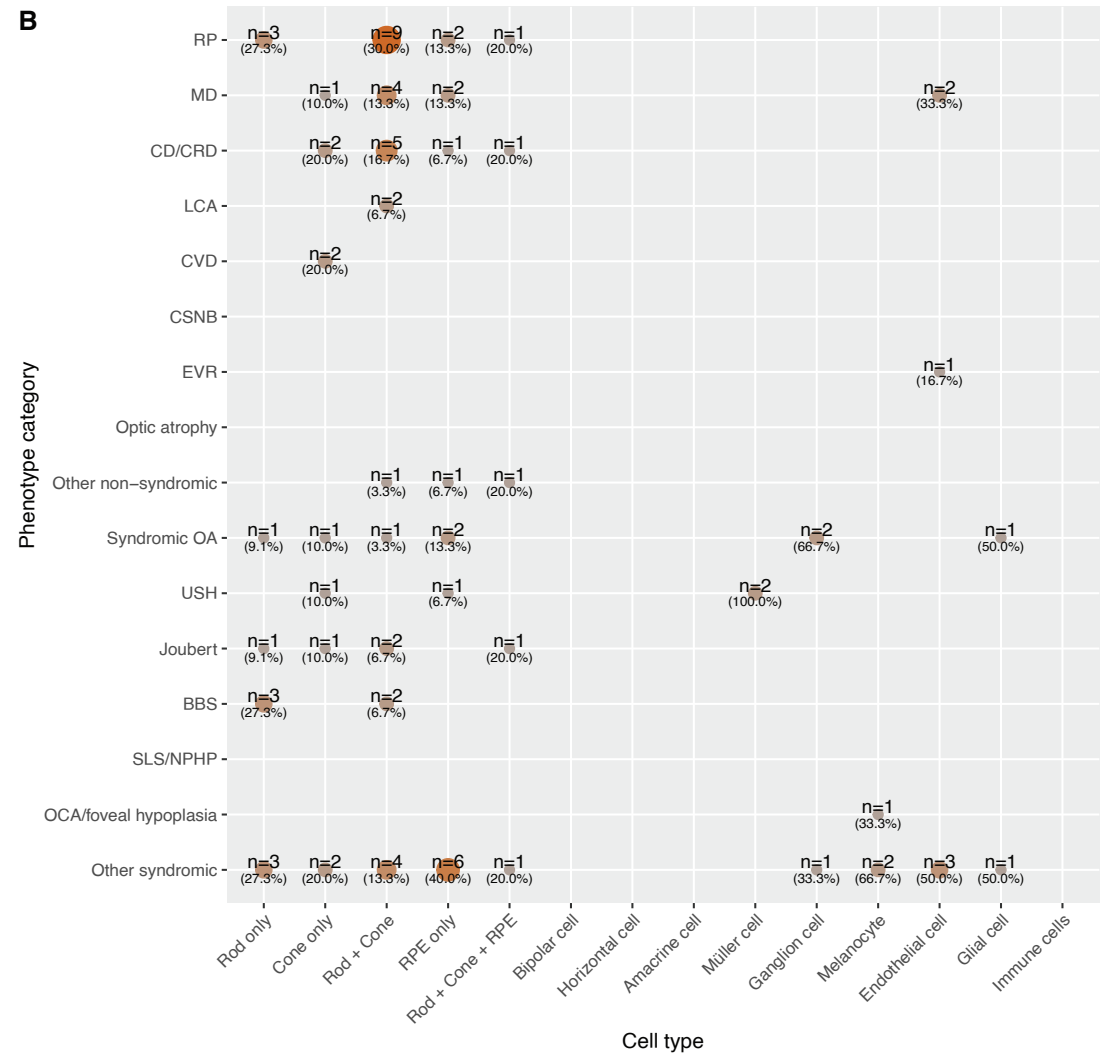

Supplement: Supplement 6 — Figure S6: Co-occurrence matrices between phenotypes and scRNAseq data. (A) Retinal-prevalent genes (also minimally expressed in other tissues). (B) Not retinal-prevalent genes, n, number of genes. [file media-6.pdf]
